# Pre-analytical sample handling effects on tear fluid protein levels

**DOI:** 10.1038/s41598-023-28363-z

**Published:** 2023-01-24

**Authors:** Marlies Gijs, Sinthuja Arumugam, Nienke van de Sande, Carroll A. B. Webers, Swaminathan Sethu, Arkasubhra Ghosh, Rohit Shetty, Jelle Vehof, Rudy M. M. A. Nuijts

**Affiliations:** 1 University Eye Clinic Maastricht, School for Mental Health and Neuroscience (MHeNs), Maastricht University Medical Center (MUMC+), P. Debyelaan 25, 6229 HX Maastricht, The Netherlands; 2 GROW Research Laboratory, Narayana Nethralaya Foundation, Bangalore, India; 3 Department of Cornea and Refractive Surgery, Narayana Nethralaya, Bangalore, India; 4 Department of Ophthalmology, University of Groningen, University Medical Center Groningen, Groningen, The Netherlands; 5 Dutch Dry Eye Clinic, Velp, The Netherlands; 6 Department of Ophthalmology, Vestfold Hospital Trust, Tønsberg, Norway; 7 Department of Ophthalmology, Zuyderland Medical Center, Heerlen, The Netherlands

**Keywords:** Biomarkers, Proteins

## Abstract

Tear fluid is emerging as a source of non-invasive biomarkers, both for ocular and systemic conditions. Accurate quantification of tear proteins can be improved by standardizing methods to collect and process tear fluid. The aim of this study was to determine sample handling factors that may influence the tear protein biomarker profile. Tear fluid was collected using Schirmer’s strips. Tear proteins were extracted by elution through centrifugation. Total protein content was determined using the bicinchoninic acid assay. Key concepts that apply to the entire sample processing cycle are tear sampling, tear storage, protein extraction and data normalization. Differences in wetting or migration length were observed between Schirmer’s strips from different manufacturers, and between protein-free and protein-rich solutions. One unit of migration length (mm) did not correspond to one unit of volume (µL). A positive correlation (r = 0.6671, p < 0.0001) was observed between migration length and total tear protein content. The most beneficial storage conditions were strips that were not stored (+ 21.8%), or underwent ‘wet’ storage (+ 11.1%). Protein recovery was the highest in 400 µL extraction buffer and independent of protein molecular weight. This study helps to explain inter- and intra-variability that is often seen with tear biomarker research. This information is critical to ensure accuracy of test results, as tear biomarkers will be used for patient management and in clinical trials in the near future. This study also highlights the need for standardization of Schirmer’s strip manufacturing, tear fluid processing and analyte concentration normalization.

## Introduction

Tear fluid is emerging as source of biomarkers^1–3^. Tear fluid consists of a mixture of proteins (of which enzymes are the majority, ± 41%^4^), lipids, carbohydrates, metabolites electrolytes, and salts. Tear fluid is rich in proteins (7–11 g/L)^5^ and tear fluid proteome studies have identified more than 3000 different tear proteins^6^. Is has been shown that tear fluid is able to represent the body (for example tear glucose levels^7^) and the brain (for example amyloid-beta and tau^8^). Since tear fluid is very easily accessible with very low-invasive sampling methods at a relatively low cost, increased interest has emerged to use it in biomarker studies.

Next to its use as a clinical tear production test, Schirmer’s strips are being used to collect tear samples for subsequent biochemical analysis. Despite its limited sample volume (typical 7–10 µL)^5^, the rise of ultra-sensitive technologies (e.g. Luminex, MesoScale, Quanterix, Olink) has led to an increasing number of papers investigating tear biomarkers. However, published data display significant variability amongst each other. Such variability could reflect biological differences among subjects as well as differences in methodology^9^. Due to low sample volume, errors can be easily introduced causing intra-variability. The variety of approaches due to limited standardization of collecting and handling is causing inter-variability^10–12^. Since variability distorts the interpretation and comparison of clinical trial outcomes, establishing Standard Operating Procedures (SOPs) will provide reliable information on tear biomarker changes^13^.

In this study, we aim to increase the understanding of the sample processing steps that can introduce inter- and intravariability in tear research as these techniques will be increasingly used for patient management (for example MMP-9 in dry eye disease^14^) as well as a primary endpoint in clinical trials.

## Results

### Factors influencing tear fluid sampling

**Figure 1:**
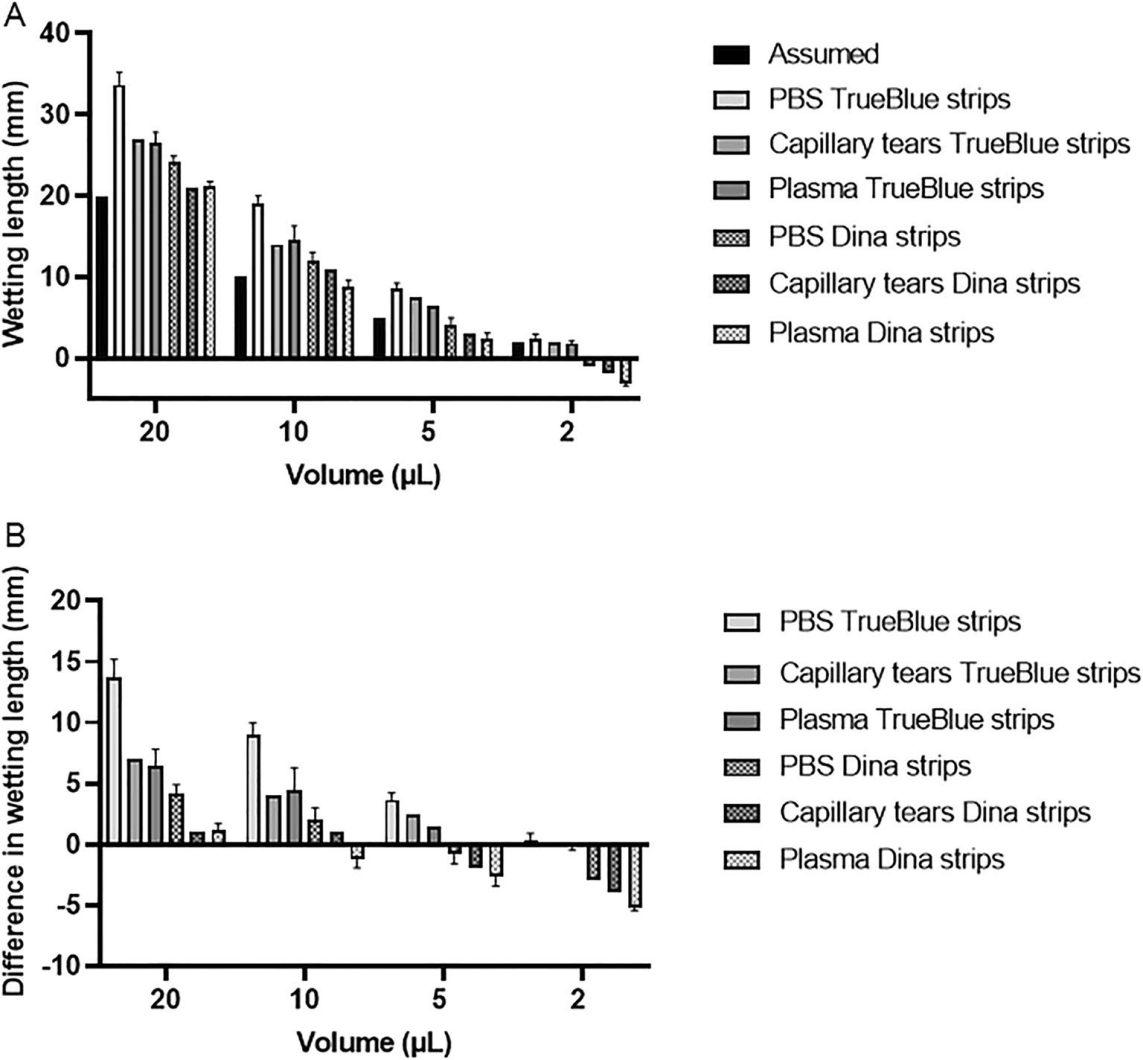
Factors influencing tear fluid sampling. (**A**) Migration length of fixed volumes of PBS or plasma on Schirmer’s strips (TrueBlue and Dina) from different manufacturers. Bars indicate mean ± SD. n = 3 for PBS and plasma, n = 1 for capillary tears, (**B**) differences in wetting length as compared to the theoretical/assumed values. Bars indicate mean ± SD. n = 3 for PBS and plasma, n = 1 for capillary tears.

We compared the tear fluid wetting or migration length on Schirmer’s strips (TrueBlue and Dina) from two different manufacturers after applying fixed volumes (2, 5, 10 or 20 µL) of PBS, capillary tears and plasma (Fig. 1A). For each of the tested volumes, the migration length on TrueBlue strips was higher than on Dina strips. The largest difference in migration length between the two strips was observed for the highest tested volume (20 µL) of PBS (difference of 9 mm) (Fig. 1B). Remarkably, after applying 2 µL of PBS, capillary tears or plasma on Dina strips, the wetting length did not reach the first (‘zero’) scale mark and we needed to estimate a negative wetting length. Differences in wetting length were also observed when comparing the migration length of equal volumes between PBS (a protein-free salt solution), capillary tears and plasma (a protein-rich biological fluid with a ten times higher total protein concentration than tear fluid, on average 60–80 g/L^15^). In Fig. 1A, it is visualized that PBS migrates further than capillary tears and plasma for each of the tested volumes on strips of both manufacturers, while the migration lengths for capillary tears and plasma are comparable. In the literature, it is often assumed that one unit of migration length (1 mm) corresponds to one unit of tear volume (1 µL)^16,17^. Our results show that this is not always true. Low volumes of PBS (2 µL) tend to migrate less than expected (− 1 mm for Dina strips only), while large volumes (20 µL) display higher migration lengths than expected (33.7 ± 1.5 mm for TrueBlue strips, 24.2 ± 0.8 mm for Dina strips) (Fig. 1A). Similar results, though less pronounced, were obtained when applying capillary tears or plasma on Schirmer’s strips.

**Figure 2:**
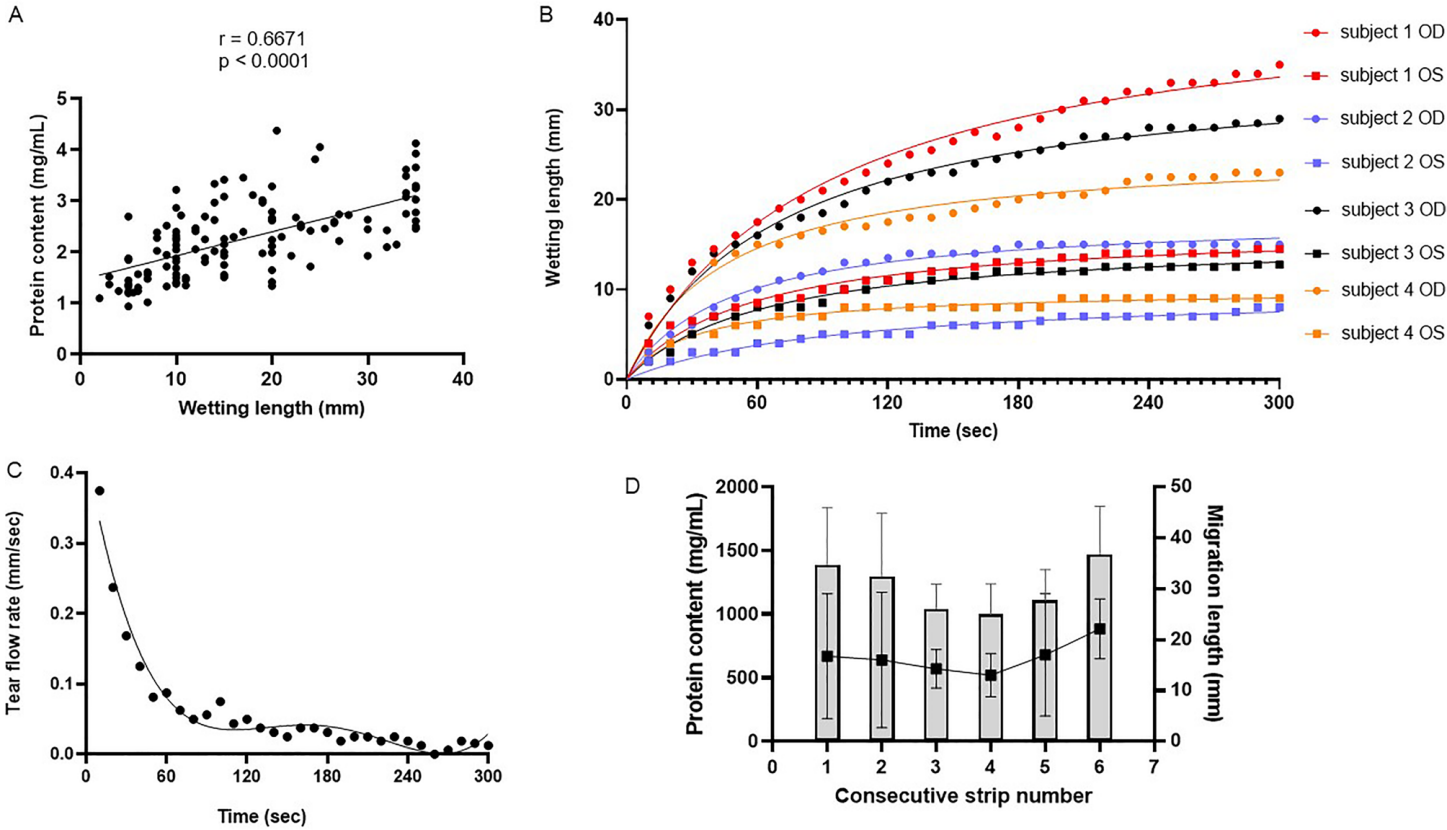
Factors influencing tear fluid sampling. (**A**) Scatter plot of total protein content versus wetting length, n = 115, (**B**) wetting length as measured each 10 s after insertion, n = 8, (**C**) average tear flow rate plotted versus time, (**D**) total protein content of consecutive sampled tears. Consecutive strip numbers correspond to the time of strip collection from baseline: strip 1 (0 min), strip 2 (5 min), strip 3 (15 min), strip 4 (35 min), strip 5 (60 min) and strip 6 (90 min). Bars and squares indicate mean ± SD. n = 4. Differences between time points were not significant (p = 0.3769).

Migration length is considered as a measure of tear fluid production. Results from 115 patient tear fluid samples demonstrate that migration length was positively correlated with total protein content (r = 0.6671, p < 0.0001 (Fig. 2A). Completely wetted strips (with a migration length of 35 mm), that are often considered reflex tears, did not have lower (diluted) protein concentrations. When recording the migration length every 10 s after application of the Schirmer’s strip in the lower conjunctival sac of eight eyes, we observed that the migration length steeply increased during the first 60 s, after which it attenuated and reached a plateau (Fig. 2B). This measurement was converted into the average tear flow rate (Fig. 2C). Tear flow rate was the highest at the first ten seconds (0.375 mm/s or 22.5 mm/min) and declined thereafter until 120 s (0.05 mm/s or 3 mm/min) when tear flow rate stabilized (0—0.05 mm/s). The effect of consecutive tear fluid sampling on the protein content and migration length was determined at several time points up till 90 min after collection of the first strip. Figure 2D shows that highest protein content was found in the sixth strip (1472.21 ± 375.6 µg/mL). Protein content slightly decreased for each consecutive sample until the fourth strip (997.4 ± 241.1 µg/mL), where after protein content increased again.

### Factors influencing tear fluid storage

**Figure 3:**
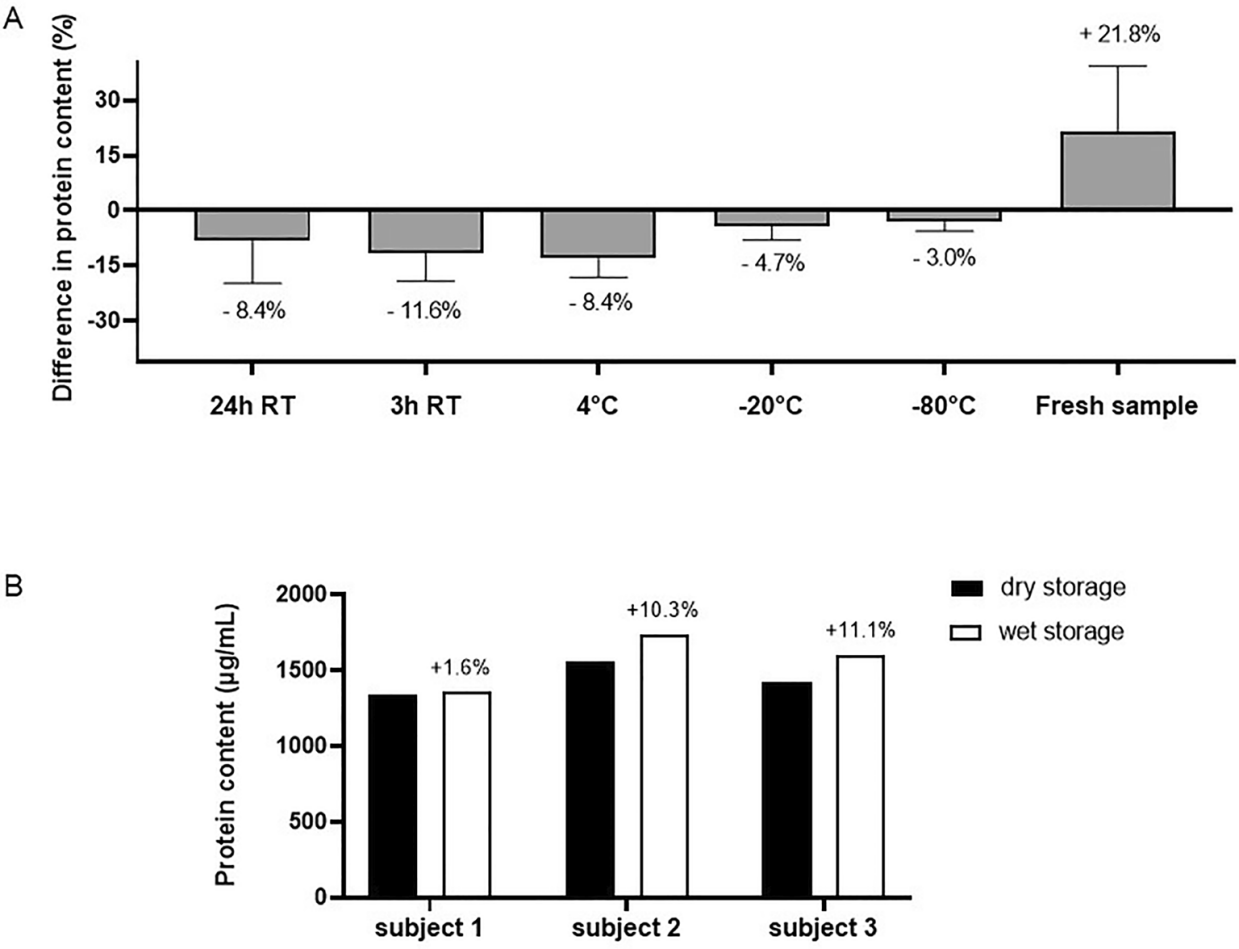
Factors influencing tear fluid storage. (**A**) Protein content of strips stored at different storage conditions, bars represent mean % ± SD change in protein content from control (= − 80 °C), n = 3, (**B**) Protein content of strips stored in wet versus dry storage conditions, bars represent individual values, labels indicate % change in protein content from control (= dry storage), n = 3 subjects. Differences between storage temperatures were not significant (p = 0.0611).

Figure 3A shows the relative percentage change in tear fluid protein concentration at different storage conditions compared to the standard (i.e. storage at − 80 °C). Our results show the beneficial effect (+ 21.7%) in total protein content for fresh tears (analyzed immediately after tear sampling) compared to tears that were stored at − 80 °C. Storage at different temperatures shows that frozen storage at − 80 °C and − 20 °C is comparable (− 3.0% and − 4.3%, respectively) and is preferred over cold storage (4 °C, 13.0% reduction in total protein content) and storage at room temperature (11.7% reduced total protein content) (Fig. 3A). Wet storage (= tear strip submerged in extraction buffer before frozen storage) was superior (average + 7.7%) over dry storage. Figure 3B shows a strong increase in tear protein concentration for subjects 2 and 3 (+ 11.1% and + 10.3%, respectively), whereas the increase for subject 1 was less pronounced (+ 1.6%).

### Factors influencing tear protein extraction

**Figure 4:**
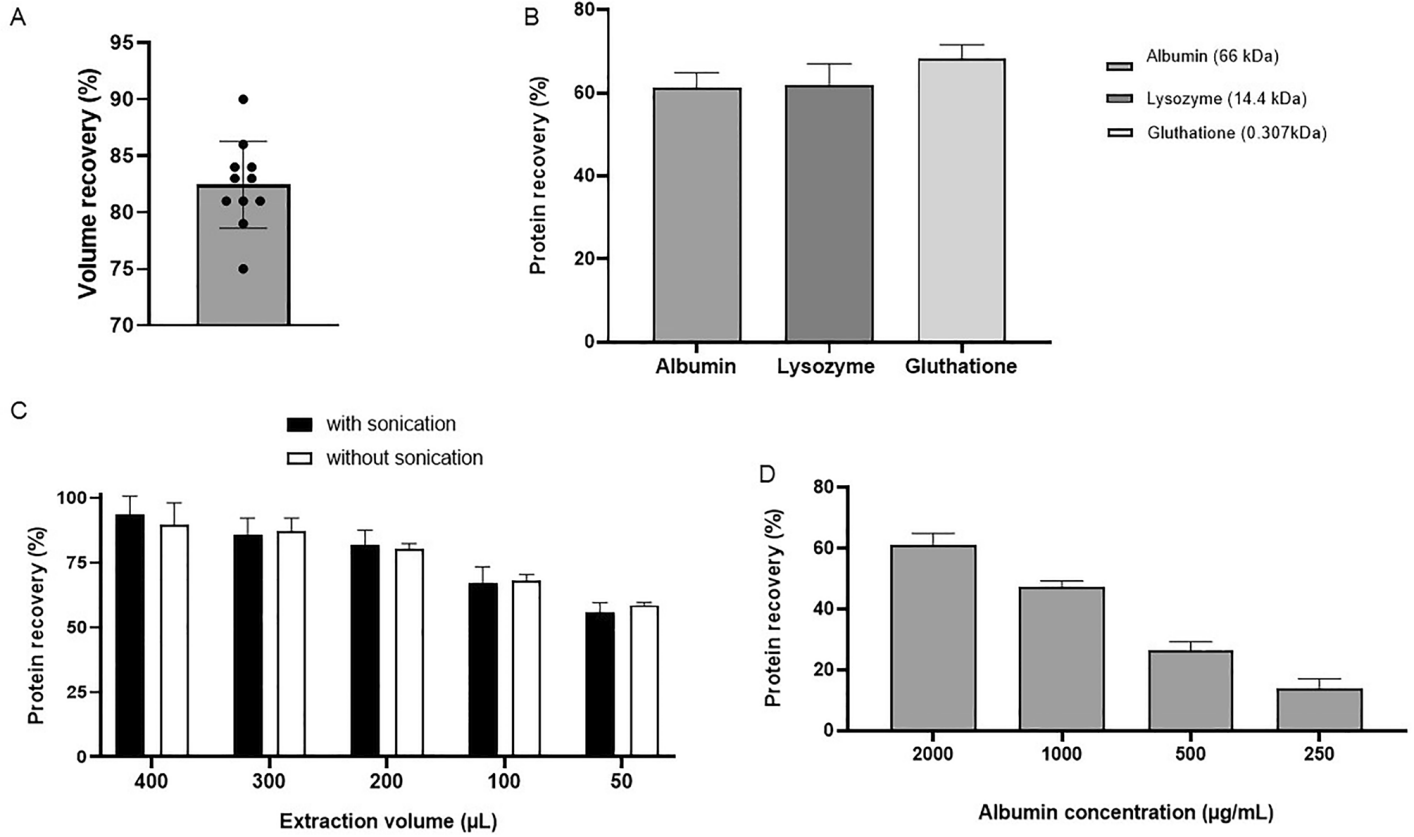
Factors influencing tear protein extraction. (**A**) Volume recovery (%) after application of a fixed volume PBS to an empty strip, n = 11, (**B**) protein recovery (%) of three proteins with different molecular weight, bars represent mean ± SD, n = 3, (**C**) protein recovery (%) of extraction performed with different extraction volumes and with and without sonication, bars represent mean ± SD, n = 3, (**D**) protein recovery (%) of extraction performed in samples with a different initial albumin concentrations, bars represent mean ± SD, n = 3.

Volume recovery, defined as the percentage of volume that can be extracted from Schirmer’s strips, is another important factor in tear biomarker research. The average volume that was recovered after application of 80 µL PBS to an empty strip was 66.2 ± 3.0 μL. This corresponds to an average volume recovery of 83% (Fig. 4A). When comparing the protein recovery of a large protein albumin (66 kDa, 61.13 ± 3.82%), and intermediate-sized protein lysozyme (14.4 kDa, 61.95 ± 5.09%) and a small three-peptide glutathione (0.307 kDa, 68.34 ± 3.36%), we did not find significant differences (p = 0.1451) (Fig. 4B). Protein recovery of albumin increased with increasing extraction buffer volumes (Fig. 4C). Protein recovery was the highest (93.56 ± 7.18%) when applying an extraction buffer of 400 µL, while using 50 µL extraction buffer resulted in the lowest protein recovery (55.94 ± 3.70%). Furthermore, the results of this test show that adding a short sonication step in the protocol did not have a great influence on the level of extracted proteins (Fig. 4C). Protein recovery was also depended on the initial protein concentration that was applied to the Schirmer’s strip. An almost linear decrease was observed for decreasing albumin concentrations, going from 61.13 ± 3.82% (2000 µg/mL) to 14.12 ± 3.08% (250 µg/mL) (Fig. 4D). Lower concentrations of initial albumin (125 and 62.5 µg/mL) were tested as well but yielded values below the limit of detection. To evaluate whether empty Schirmer’s strips yield a background signal in protein quantification assays, a BCA test was performed after applying and extracting 20 µL PBS on a Schirmer’s strip (TrueBlue). We found that there was a signal corresponding to an intrinsic protein content of 3.51 μg per strip (data not shown).

## Discussion

**Figure 5:**
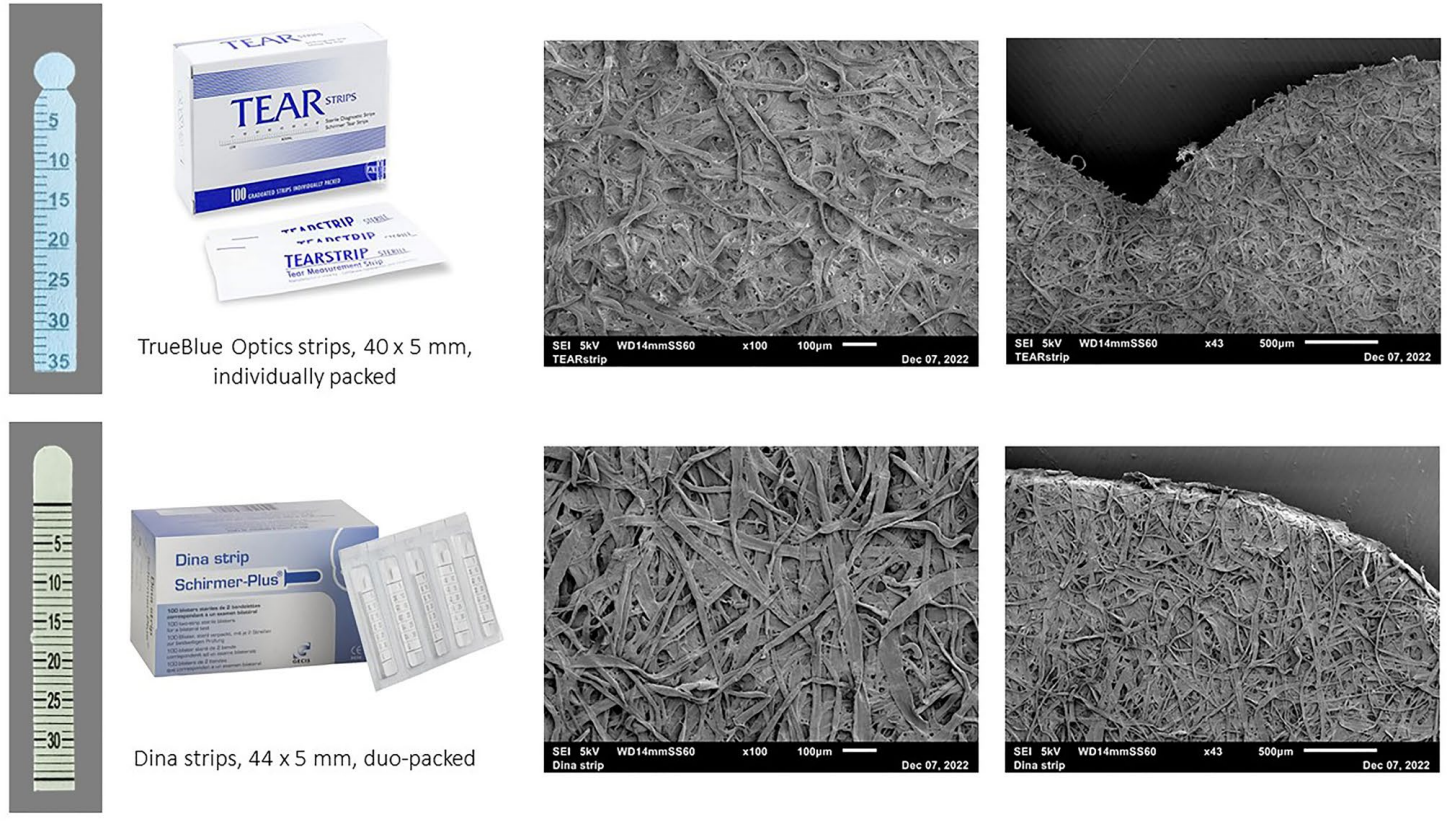
Schirmer’s strips (TrueBlue TEAR strips and Dina strips) Illustration and SEM images of the Schirmer’s strips from different manufacturers that were used in this study.

The lack of a standardized method for tear protein research makes interpretation and comparison of results difficult. In the present study, we report various factors influencing tear protein research. Firstly, we identified variables in the manufacturing of Schirmer’s strips as influencing factors of tear sampling. We noticed a difference in migration length when applying fixed volumes onto strips from different manufacturers. This suggests that the preprinted scale bar on Schirmer’s strips is not calibrated. There are multiple reasons why there can be a difference between strips from different manufacturers. One of the reasons can be a different material of the strip. The U.S. Food and Drug Administration (FDA) defines a Schirmer’s strip as ‘a device made of filter paper or similar material intended to be inserted under a patient's lower eyelid to stimulate and evaluate formation of tears’^18^. This suggests that there is no regulation of the material of the strip. Most often, strips are made of Whatman no 41 filter paper^19^. Since different materials can have different thickness, fiber densities and pore sizes, and thus different water absorption rates, this can influence the tear migration distance. Using SEM we observed larger pore sizes and less densely packed cellulose fibers for the Dina strips compared to the TrueBlue strips (Fig. 5). There seems no regulation on the dimensions of the strip, since we observed strips with different shapes. The TrueBlue strips end at 40 mm while Dina strips end at approximately 44 mm (Fig. 5). Most probably, this size difference explains why TrueBlue strip gave higher migration lengths compared to Dina strips in our experimental set-up (Fig. 1). Manufacturers likely often choose the dimensions and shape based on ease of application of the strips. For example, TrueBlue strips have rounded tips and notches to increase the ease placing the strip in the eye. This results in different surface areas of the blank tips and will lead to different absorbed volumes even before it reaches the preprinted scale bar. García-Porta et al. found that there is also a difference in weight between different strips and that thicker strips have a slower migration, but more fluid is taken up^9^. Lewin et al. demonstrated significant differences in wetting length between strips from different manufacturers^20^. Variability between manufacturers is important in tear fluid research (when normalizing protein results for migration lengths) and even more important when using migration lengths as a measure of tear production in regular care (DED diagnosis, monitoring) and in (multicenter) clinical trials. Therefore, there is an urgent need for standardization of the manufacturing of Schirmer’s strips. Until now, it is highly recommended to use Schirmer’s strips from the same manufacturer over time.

The fact that there is no standardization is also suggested from our results that demonstrate that one unit of migration length (1 mm) does not correspond to one unit of volume (1 µL). One of the reasons for this is because tear fluid that is absorbed on the head of the Schirmer’s strip (that is in direct contact with the lower eyelid) is excluded in the scale bar. But even when a Schirmer’s strip would be calibrated for one solution, it may yield different results for other solutions. Our results show differences in migration length between protein-free and protein-rich solutions. In practice, this might result in differences between tears with a low protein content and a high protein content. In contrast to what we expected, our results show a positive correlation between tear migration length and protein content. Similar positive correlations were observed by Boerger et al.^10^ Low protein containing tears seem to migrate more than tears with high protein content. Tears of 35 mm (often referred to as reflex tears) were not diluted and tears with migration length below 5 mm (typically from dry eyes) were not highly concentrated. Nevertheless, differences in protein content may influence interpretation of protein analysis results. Because of this reason, it is suggested to normalize protein results to the total protein content. Alternatively, tears of the same migration length could be collected (e.g. by removing the Schirmer’s strip from the eye after 10 mm has wetted, independent of time^16^) or by analyzing the same strip lengths (e.g. only the first 10 mm) in the lab.

The low sample volume of tear fluid may hold back clinicians and researchers to start investigating tear fluid. In view of this, we investigated the protein content of tear samples that were collected in a consecutive manner. Our results show that introducing a second Schirmer’s strip immediately after the first one does not really affect the total protein content. Tear samples do start to display lower protein concentrations after 15 and 35 min, but also restore quickly. Our results thus show that the protein content in consecutive samples remains acceptable. This is especially interesting when collecting multiple strips from one patient in order to examine very low abundant protein biomarkers or to combine various analytical techniques (such as protein and RNA analysis). Backhuber et al. investigated consecutive sampling by means of inserting one Schimer’s strip in one eye, giving approximately 21 min equilibration time in between, followed by a second Schirmer’s strip in the contralateral eye. In their setting, they observed that consecutive collection significantly influenced tear flow rates, IgG, and protein concentrations^21^. We also investigated the tear flow rate (mm/s) and observed a decreasing trend. We believe that this observation is dimply due to the initial absorption of the basal tear that is present on the ocular surface (7–10 μL) followed by absorption of newly produced tear fluid (1–2 μL/min).

Secondly, we investigated factors influencing tear storage. Storage is highly desired in order to wait until samples from all patients have been collected and to perform the analysis with minimal batch and lot variations. However, it has been shown that long-term storage (> 4 months at − 70 °C) reduced total tear protein concentration^22^. Our results show higher total protein content of fresh tears compared to frozen tears. However, the analysis of fresh tears is practically unfeasible, unless for using a point of care test that does not require storage, but immediate analysis. As expected, lower temperatures yield higher protein contents. Hamm-Alvarez confirmed no significant loss of signal detected for any protein of interest after storage for 1 or 2 weeks at − 80 °C^23^. However, availability of a − 80 °C freezer is limited in most settings, especially in local hospitals, GP practice, outpatient testing facilities or home visits. For such settings, the usage of standard freezers (− 20 °C) is advised. Storage of strips at temperatures ≥ 4 °C easily lead to protein degradation and/or inactivity. This may be especially true for tear fluid due to its high content of enzymes (± 41%)^4,24^ in order to protect the ocular surface from various pathogens and the inherent instability of certain cytokines and chemokines^25,26^.

Additionally, we demonstrate that ‘wet’ storage, where the collected Schirmer’s strip is submerged in PBS buffer containing a protease inhibitor cocktail, was beneficial as compared to ‘dry’ storage. PBS is a water-based salt solution that is the ideal environment for protein dynamics, stability and structure. The addition of a small volume (100 µL) PBS during storage seems to be beneficial, potentially because of the larger aqueous environment and/or dilution of the highly concentrated proteins. Furthermore, the addition of protease inhibitors might prevent protein degradation during storage. The use of pre-filled buffer tubes to store Schirmer’s strips immediately after tear sampling is however complicated by the storage temperature (4 °C) and limited shelf-life (1–2 weeks) of the protease inhibitors, as well as the dilution of your protein of interest. Nevertheless, this ‘wet’ storage condition might be of interest for well-defined small-scale clinical studies. Storage as a dried (unfrozen) strip, in analogy with dried blood spots, has not found its applications for tear fluid yet, although it removes the burden of frozen storage and shipping. Presumably because those tests primarily focus on DNA and RNA analysis, rather than protein analysis.

Thirdly, our results present ways to optimize the protein extraction method. Suboptimal recovery of tear proteins and volume is inherent due to the absorptive material of a Schirmer’s strip. Similarly, Denisin et al. identified two major sources of protein loss: sample handling and retention of protein on the Schirmer’s strip^19^. In the same way, Bertram et al. showed that volume recovery from Schirmer’s strips is suboptimal^16^. Nevertheless, our results show that, when protein extraction was performed in 400 µL buffer, protein recovery was nearly 100%. Higher buffer volumes would therefore not be needed to improve protein recovery. However, such high buffer volumes will dilute the sample and the protein(s) of interest, considerably. The choice of the optimal buffer volume critically depends on the abundance of the protein of interest as well as the required sample volume for the analysis technique. Standard ELISA’s usually require between 25 and 100 µL of sample, the immunoassays of Mesoscale require 25 µL sample volume, whereas Quanterix assays usually needs a minimum of 110 µL as it includes a significant amount of dead volume. If necessary, one might consider using lower buffer volumes with acceptable protein recovery rates (e.g. 300 µL), or using analysis techniques that measure multiple analytes in one sample (e.g. multiplex immunoassays and proteomics).

We expected that the level of protein recovery would be dependent of the protein molecular weight. Surprisingly, our results show a similar protein recovery for a large protein (albumin, 66 kDa), a medium-size protein (lysozyme, 14.4 kDa) and a small three-peptide (glutathione, 0.307 kDa). Other factors, such as protein hydrophilicity or surface charge, may play a role. Previous studies showed that some proteins, including interleukins and g‑interferon, bind tightly to tear sponges, making the recovery and extraction of these proteins more difficult^19,27^. However, Denisin et al. showed that the extent of proteins retained on the Schirmer’s strip and lost during sample handling is not associated with protein surface charge or surface hydrophilicity for the spectrum of proteins that they studied^19^.

Our results show that extraction of an empty Schirmer’s strip yields background signal in protein quantification assays. We hypothesize that cellulose paper and/or the ink from the scale bar contain a small number of peptides or proteins that are extracted together with the tear proteins. Because of this, it is important to always use the entire Schirmer’s strip, and not only the wetted part, in the extraction protocol.

The second reason to always use the entire Schirmer’s strip in protein analysis is because we expected that tear proteins are differently distributed within the strip, as large and sticky proteins are less likely to migrate together with the tear fluid through the paper fibers. Our results confirm that the highest protein content was found in the 0–5 mm region of the Schirmer’s strip. This was also due to the size of this part of the Schirmer’s strip that was relatively larger when compared to every other 5-mm of the strip as it also contained the head of the strip. Secondly, the head of the strip is in direct contact with the conjunctiva and may therefore contain additional cellular proteins from conjunctival cells. Remarkably, the protein distribution within the Schirmer’s strip does not seem to differ as similar protein concentration were found in each 5-mm pieces, both for tear fluid collected from subjects as for individual protein solutions. We speculated that small peptides would migrate easily together with the tear flow and pile up at the end of the strip, but our results show no differences in protein (and peptide) concentration within the Schirmer’s strip. These insights in the distribution profile of tear proteins are important for technologies that only use part of the strip for protein analysis. For example, when a punch of the strip is made, similarly to dried blot spot test, for subsequent protein analysis (Olink) or DNA analysis. Our results also suggest that, if most proteins are concentrated in the first 0–5 mm, tears from patients with dry eyes syndrome or Sjogren’s disease are still worth investigated despite the usually very low samples volumes, and that high tear volumes are not a prerequisite for protein biomarker analysis.

Next to the methods of tear sampling, storage and extraction, processing of the raw data does not seem to be standardized yet. A variety of data normalization methods are available in the literature. We looked into various data normalization methods and listed each advantages and disadvantages (Table 1). Also here, standardization would allow better to compare results of different publications with each other, and subsequently to draw conclusions about the disease pathophysiology. For other body fluids, there seems to exist more consistency. For example, urinary biomarkers are most frequently normalized to urine creatinine levels to control for variations in urine flow rate^28^. We believe that normalization to an endogenous tear protein, such as lactoferrin or lysozyme, may be the most preferred option for future tear protein research, although it requires a part of your (already small) extracted tear volume and comes with additional costs. Although lactoferrin is a regulated protein and its concentration remains almost constant^29^, this situation may only be true in a healthy situation.

**Table 1:** Factors influencing protein data normalization.

Tear protein normalization method	Advantages	Disadvantages
No normalization (raw data)	Easy	Raw data depends on extraction buffer volume
Based on tear volume/migration length	The migration length is readily available from the patient file Corrects for reflex tears	One unit of migration length does not correspond to 1 µL (see Fig. 1) Does not work for low migration lengths of zero or negative values
Based on tear weight or dry weight of extracted tears	Corrects for reflex tears	Requires a (very) sensitive balance Needs to be weighted immediately after collection/before freezing Weighing requires time at ambient temperatures introducing protein stability issues
Based on total protein content	Corrects for reflex tears Allows to compare different body fluids with each other	Requires part of extracted tear volume Need for additional protein assay (and associated costs) Dissipates pathological situations with high protein content (e.g. inflammation)
Based on ratio with an endogenous protein level, e.g. lactoferrin or lysozyme	Allows to compare different body fluids with each other Established method for other body fluids (e.g. creatinine in urine)	Requires part of extracted tear volume Need for additional protein assay (and associated costs) Changes in endogenous proteins are often associated with diseases (e.g. DED)

Summary of advantages and disadvantages of different tear protein normalization methods.

In this study, we limited our investigation to looking at the overall protein content. Therefore, the results may differ when looking at individual proteins of interest, due to differences in protein size, hydrophilicity and abundance. Another avenue that is worth investigating in the future is the effect of the above factors on protein activity and three-dimensional (3D) structure. For certain proteins, for example alpha-secretase^30^, its activity rather than its presence in a biological fluid may be a more suitable biomarker. Additionally, preservation of the original 3D structure of proteins is essential for antibody recognition and binding in immunoassays. In case of issues, one might turn towards mass spectrometry analysis that digest proteins into peptides irrelevant of its 3D structure. Specifically for proteomic sample preparation of Schirmer’s strips, in strip digestion has been suggested to yield higher protein levels^6,31^.

In conclusion, despite the increasing popularity of the use of tear proteins as biomarkers, the field seems to lack standardization for tear sampling, storage and handling. Although often overlooked in the research laboratory, carefully controlled and well-characterized tear sample preparation is critical in yielding scientifically and clinically meaningful results. With this study, we provided insights in the factors that affect tear proteins during processing in the lab as tools to set up guidelines. Tear protein biomarkers still have to become key tools during the clinical decision-making process and standardization of the protocol is the first step towards that.

## Methods

### Tear samples

Tear samples were available from healthy subjects from the Biobank TearFluid Maastricht, University Eye Clinic Maastricht, Maastricht University Medical Center (MUMC+) and from a previously completed clinical trial involving healthy volunteers^32^. The local ethical committee of the Maastricht University Medical Center (MUMC+) approved the study protocols and these studies followed the tenets of the current version of the Declaration of Helsinki. Written informed consent was obtained from all subjects.

### Tear fluid sampling

**Figure 6:**
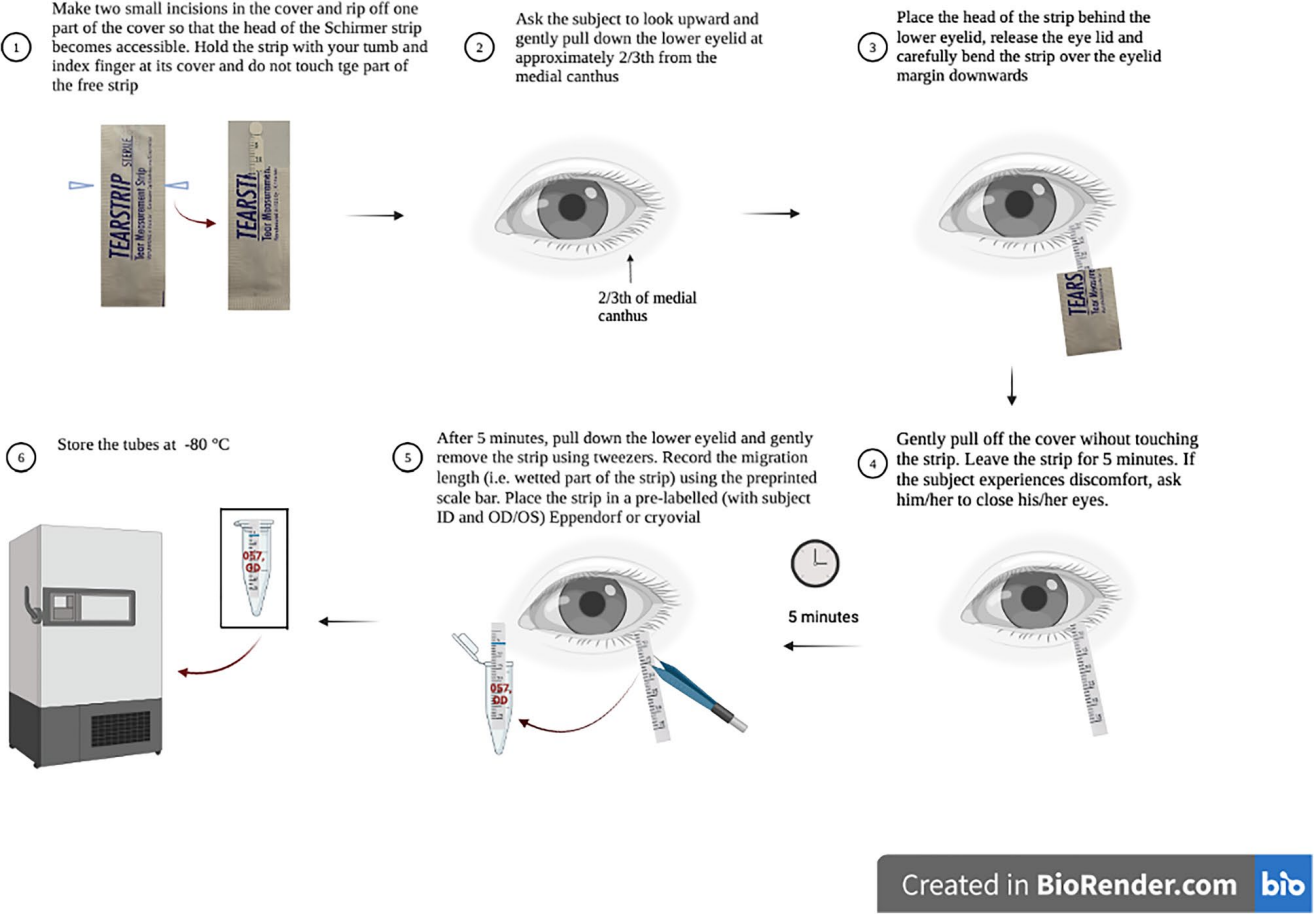
Tear fluid sampling protocol. Tear fluid was collected from the left and right eye without topical anesthesia. Care was taken not to touch the strip with fingertips. The migration length was read after 5 min from the preprinted scale bar on the strips. Immediately after sampling, samples were stored at − 80 °C, unless noted otherwise. Image created in Biorender.com.

A detailed workflow of the tear sampling method is shown in Fig. 6. Schirmer’s strips from TrueBlue Optics (Contacare Ophthalmics and diagnostics, Gujarat, India) were used throughout the study. Dina Schirmer’s strips (Tear Schirmer Plus, GECIS, Sarl, France) were used for the manufacturer comparison study (Fig. 5). Tear fluid was collected from the left and right eye without topical anesthesia. Care was taken not to touch the strip with fingertips. The migration length was read after 5 min from the preprinted scale bar on the strips. In case of completely wetted strips (35 mm), strips were removed < 5 min. Immediately after sampling, samples were stored at − 80 °C, unless noted otherwise. Capillary tears were collected from the inferior tear meniscus using a 10 µL glass microcapillary pipet (Kimble, Sigma-Aldrich). Following collection, the capillary tear was immediately transferred to a sterile Eppendorf tube for storage at − 20 °C.

### SEM images

Schirmer strips were coated with gold using a 108 Auto Sputter Coater (TED PELLA inc., Redding, CA, USA) on auto settings for 30 s. Hereafter, scanning electron microscopy (SEM) was performed using a JSM- 6010PLUS/LA Analytical Scanning Electron Microscope (JEOL Europe, Nieuw-Vennep, The Netherlands). The images were made with a magnification of 43 × and 100 × at 5 kV in SEI mode.

### Protein extraction

**Figure 7:**
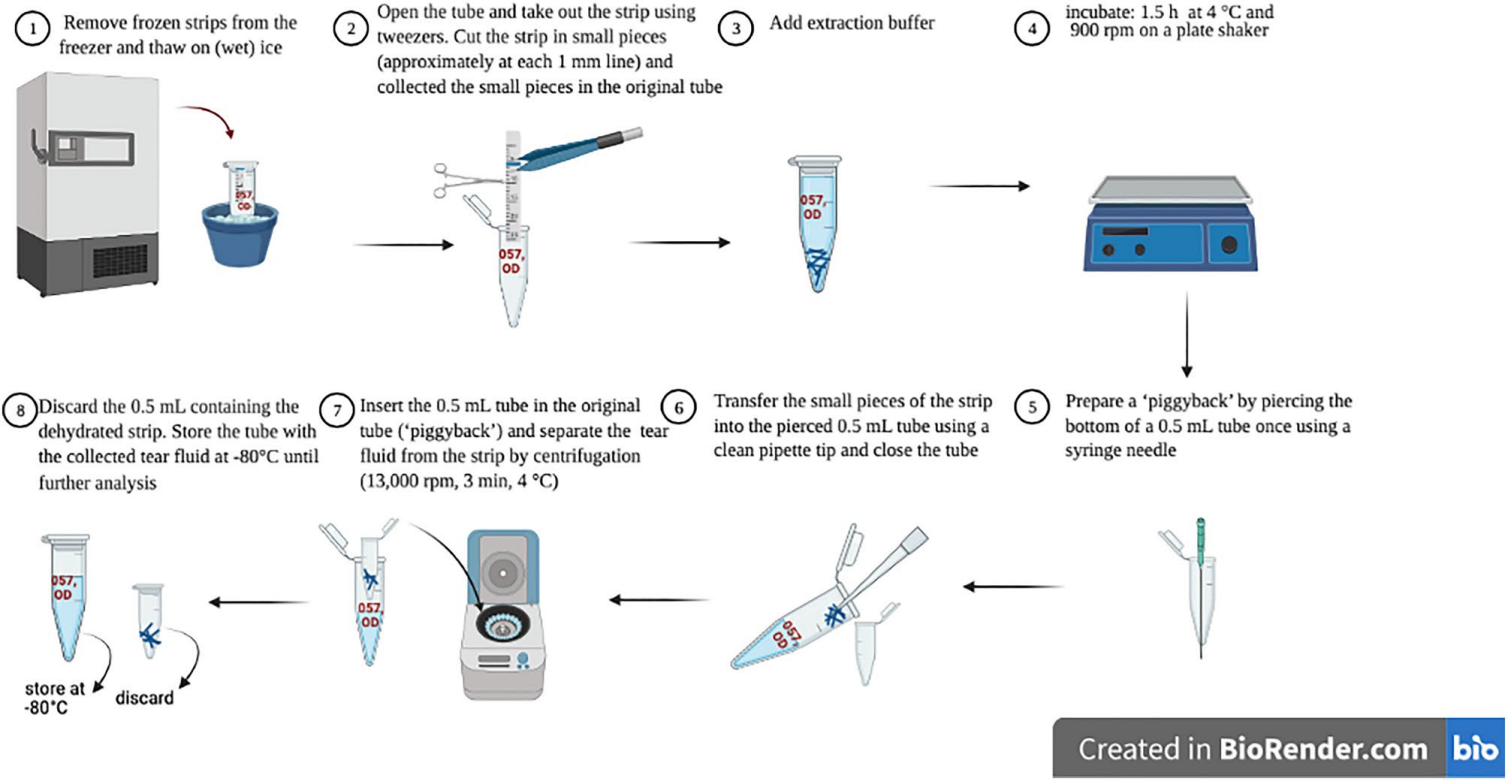
Tear fluid extraction protocol. Tear fluid was extracted from the Schirmer’s strip by agitating small cut pieces of these strips in a volume of extraction buffer at 4 °C for 1.5 h. Tear fluid was then eluted by centrifugation (‘piggyback method’) and stored at − 80 °C until further use. Image created in Biorender.com. The label “057 OD” illustrates an example patient ID and right eye.

A detailed workflow of the tear protein extraction method is shown in Fig. 7. Briefly, tear fluid was extracted from the Schirmer’s strip by agitating small cut pieces of these strips in a volume of phosphate-buffer saline (PBS, pH 7.4), and cOmplete™ Protease Inhibitor Cocktail (Roche, Basel, Switzerland) at 4 °C for 1.5 h^33^. Tear fluid was then eluted by centrifugation (‘piggyback method’) and stored at − 80 °C until further use.

### Protein quantification

The total protein content was determined using the bicinchoninic acid (BCA) Protein Assay Kit (Pierce™, Thermo Fisher Scientific, Waltham, USA) according to the manufacturer’s instructions. Absorbance was measured at 562 nm on a microplate reader (CLARIOstar PLUS, BMG Labtech, Germany).

### Tear sampling experiments

To investigate the difference in migration length between Schirmer’s strips of different manufacturers, fixed volumes (range 2–20 µL) of PBS, capillary tears or plasma (rabbit plasma w/ EDTA, Biowest, Nuaillé, France) were gently pipetted (in a single time addition) onto the head of the Schirmer’s strip that was held vertically. The migration length was recorded from the Schirmer’s strips when the solution did not migrate any further. Results are expressed as mean migration length (mm) and as difference (mm) from the theoretical migration length. Each condition was investigated in triplicate (n = 3) for PBS and plasma, and in singlicate (n = 1) for capillary tears. All subsequent experiments were performed using the TrueBlue Schirmer’s strips only.

To investigate the correlation between migration length and protein content, a total of 115 tear samples were (previously) collected, stored, extracted in 60 μL PBS + 25 × cOmplete, and quantified as described above.

The tear flow rate was determined by placing a Schirmer’s strip in the eye of a subject and recording the migration length every 10 s for a period of 5 min. Results are expressed as individual migration length (mm) and as mean tear flow rate (mm/s). This experiment was performed on four subjects, two eyes per subject (n = 8).

To investigate the effect of consecutive tear fluid sampling on the total tear protein concentration, six Schirmer’s strips were collected at distinct time points (range 0–90 min). After storage, tear proteins were extracted from the strips in 100 μL PBS + 25 × cOmplete, and quantified as described above. Results are expressed as mean tear protein concentration (µg/mL). The experiment was performed on two subjects, two eyes per subject (n = 4).

### Tear storage experiments

To investigate the tear protein stability in different storage temperatures, immediately after tear fluid sampling, the strips were cut vertically into two evenly spaced pieces. One part was stored at − 80 °C (control) and the other part was stored at different temperatures (ranging from room temperature (RT) to − 80 °C) or extracted immediately (‘not stored’). After storage, tear proteins were extracted from the strips in 100 μL PBS + 25 × cOmplete, and quantified as described above. Results are expressed as relative percentage change compared to the control (− 80 °C). Each condition was investigated in triplicate (n = 3).

To investigate the tear protein stability in different storage conditions, immediately after tear fluid sampling, one strip was stored in an empty 1.5 mL Eppendorf tube (‘dry storage’), whereas the other strip was placed in a 1.5 mL Eppendorf tube prefilled with 100 μL PBS + 25 × cOmplete (‘wet storage’). Both tubes were stored at − 80 °C. After storage tear proteins were extracted and quantified as described above. Results are expressed as relative percentage change compared to the control (‘dry storage’). The experiment was repeated in triplicate (n = 3).

### Tear protein extraction experiments

To investigate the volume recovery, extraction of an empty Schirmer’s strip was performed as described above in 80 μL PBS buffer. The eluted volume that was centrifuged out of the strip was measured by pipetting. Results are expressed as percentage recovery from the original volume. The experiment was repeated 11 times (n = 11).

The effect of buffer volume and ultrasonication on the protein extraction efficiency was investigated by pipetting 35 μL of the albumin stock solution onto the head of a Schirmer’s strip. Samples were stored at − 80 °C. Subsequently, protein extraction was performed in different buffer (PBS + cOmplete) volumes, ranging from 50 to 400 µL. The effect of sonication was investigated by submerging the samples for 5 min in a sonication water bath prior to elution by centrifugation. Results are expressed as percentage recovery from the original stock solution. Each condition was repeated in triplicate (n = 3).

The effect of protein concentration on the protein extraction efficiency was investigated by applying different concentrations (ranging from 2 mg/mL to 62.5 µg/mL) of the albumin stock solution onto the head of a Schirmer’s strip. After storage, proteins were extracted in 100 μL PBS + cOmplete buffer and quantified as described above. Results are expressed as percentage recovery from the original stock solution. The experiment was repeated in triplicate (n = 3).

### Data analysis

Statistical and graphical analysis was done using GraphPad Prism version 9 (GraphPad Software, San Diego, CA, USA). Data are expressed as mean ± SD. Correlation between protein content and migration length was assessed using Spearman correlation analysis. Differences between consecutive samples and storage temperatures were analyzed by one-way ANOVA with Kruskal–Wallis testing. Figures 5 and 7 were created in Biorender.com.

## Data Availability

The datasets used and analyzed during the current study are available from the corresponding authors on reasonable request.

## Abbreviations

SOP: Standard operating procedure
PBS: Phosphate-buffered saline
SD: Standard deviation
BCA: Bicinchoninic acid
kDa: Kilo Dalton
DED: Dry eyes disease
